# Clinical trials in the Eastern Mediterranean Region: a ten-year landscape analysis

**DOI:** 10.7189/jogh.16.04287

**Published:** 2026-09-25

**Authors:** Shrouk M Abdou, Akbar Fotouhi, Arshad Altaf, Ghassan Karam, Arash Rashidian

**Affiliations:** 1 Department of Science, Information and Dissemination, World Health Organization Eastern Mediterranean Region, Cairo, Egypt; 2 Department of Epidemiology and Biostatistics, School of Public Health, Tehran University of Medical Sciences, Tehran, Iran; 3 Department of Research for Health, World Health Organization, Geneva, Switzerland

**Keywords:** clinical trials, Eastern Mediterranean Region, health research systems, trial registries, health policy, research governance, research equity

## Abstract

**Background:**

In 2022, the World Health Organization (WHO) called on countries to strengthen their capacities for the conduct of impactful clinical trials and generating high-quality evidence on health interventions. The Eastern Mediterranean Region (EMR) faces challenges in this regard, with research output concentrated in a few countries and institutions.

**Methods:**

We analysed the registration data of clinical trials from the WHO International Clinical Trials Registry Platform for all 22 countries and territories of the EMR from 2013 to 2023. Following data cleaning, deduplication, and intervention classification, we descriptively analysed the trials across time, countries, intervention types, phase, sample size, sponsorship, and participant demographics.

**Results:**

We identified 49,529 interventional trials, representing a three-fold increase in annual registrations over the decade. Most studies conducted in 2023 originated from only a few countries: Iran (57%), Egypt (23%), and Pakistan (14%), followed by Saudi Arabia (1.5%), Iraq (1.4%), and Tunisia (1.2%). Despite the increase in absolute numbers, the proportion of clinical trials with pharmacological interventions declined from 61.5% in 2013 to 42.4% in 2023. While the findings demonstrate significant growth in regional clinical trial activity, they also highlight persistent challenges, as interventional studies with small sample sizes remain common.

**Conclusions:**

Although our analysis reveals substantial growth in clinical trial registrations across the EMR over the past decade, persistent disparities in research capacity remain, while the role of industry remains limited. Coordinated efforts are required to increase multi-centre and multi-country studies in order to address the key health priorities and contribute more effectively to the development of solutions that address regional needs.

In 2022, the World Health Organization (WHO) called on countries to strengthen their capacities for the conduct of impactful clinical trials and providing high-quality evidence on health interventions [1,2]. Achieving this, however, would require improvements in research capacity, governance, and coordination across the globe, considering the finding and recommendations of the independent panel of pandemic preparedness and response in its report entitled ‘COVID-19: make it the last pandemic’ [2].

The need to expand such capacities in the Eastern Mediterranean Region (EMR), which comprises 22 countries and territories from Pakistan to Morocco, is evident. While the WHO Advisory Committee on Health Research emphasised the importance of capacity building in large-scale research studies for health in the region, the progress in research output remains slow and limited to a few countries and institutions. Specifically, a review of publications found that three academic institutions accounted for over 10% of all publications led by investigators from the region, while the 25 most prolific EMR institutions collectively accounted for 44% of all biomedical and health research publications in EMR [3]. The COVID-19 pandemic further highlighted the challenges of responding to evidence needs in a timely manner in the region [4,5].

Besides obtaining ethical clearance, all clinical trials (CTs) need to be registered in an accredited, publicly accessible registry. One such registry is the WHO’s International Clinical Trials Registry Platform (ICTRP), which, since 2005, collates the data from all other global registries. Analysing trial data from these registries is particularly valuable because only a portion of trials result gets reported in peer-reviewed publications [6].

The capacity of organisations in the EMR to conduct of clinical trials and the landscape of clinical trials activity has not been adequately mapped. Previous studies have been country-focused or limited in their time spans: Feizabadi *et al*. covered the clinical trials registered from Iran up to 2015 [7], Siddiqui *et al*. focused on Pakistan up to 2019 [8], and Al-Hajri *et al*. documented clinical trial registrations from Bahrain, Kuwait, Oman, Saudi Arabia, and the United Arab Emirates up to 2019 [9]. Clinical trials registered from other countries in the region (*e.g.* Egypt) were only covered in other multi-country assessments [10].

We address this gap by consolidating and analysing clinical trial registration data available on the WHO ICTRP from all countries in the Eastern Mediterranean Region (EMR) from 2013 to 2023, with the aim of providing a foundational mapping of the region’s clinical research landscape to inform future capacity-building and governance efforts. The analysis will cover key features including trends over time, geographical distribution, intervention types, target sample size, trial phase, sponsorship, participant demographics, and recruitment status.

## METHODS

We analysed trial registration data from the ICTRP for all 22 EMR countries between 1 January 2013 and 31 December 2023. The retrieved .csv ICTRP dataset included 24 different variables, from which we include the following: ‘scientific name’, ‘intervention type’, ‘target size’, ‘gender’, ‘age’, ‘registration date’, ‘allocation’, ‘primary purpose’, ‘primary sponsor’, and ‘bridged type’. We consequently use the term ‘clinical trials’ in accordance with the ICTRP classification [6], which defines interventional studies as any study in which participants are prospectively assigned to an intervention. This definition extends beyond pharmaceutical drug trials to encompass behavioural, physical, device-based, and other intervention types.

### Data preparation

We excluded records without registration dates and observational studies, retaining only interventional studies. The remaining records underwent a structured three-step deduplication process. First, we identified trials that were registered multiple times across platforms and shared the same trial ID, collapsing them into a single entry using the ‘bridged type’ field. Second, we removed trials sharing the same trial ID within the same registry. Third, we flagged trials with similar identifying details for manual review, and merged those confirmed to represent the same study. Records for which duplicate status remained ambiguous were retained in the data set. Missing values in categorical fields such as ‘phase’, ‘sponsor type’, and ‘gender’ were retained as a separate ‘not reported’ category rather than imputed, to avoid introducing systematic bias.

We classified intervention types using the ICTRP intervention type field, followed by a manual check to verify and correct ambiguous entries. This process yielded eight categories: ‘behavioural’, ‘biological’, ‘device-based’, ‘pharmacological’, ‘physical’, ‘herbal’, ‘radiation’, and ‘vaccine’. Behavioural interventions included smoking cessation programmes, dietary modifications, and cognitive-behavioural therapy. Biological interventions involved biological products or modifications, including gene therapy and stem cell therapy. Device-based interventions included medical equipment such as pacemakers, insulin pumps, prosthetics, and surgical implants. Pharmacological interventions relied on medications such as antibiotics, chemotherapy, and hormone therapies. Physical interventions involved manual or therapeutic techniques, including physiotherapy, acupuncture, and chiropractic care. Herbal interventions involved the use of plant-based remedies, such as medicinal herbs, extracts, and traditional herbal formulations. Radiation interventions employ radiation-based treatments such as radiotherapy for cancer, x-rays, and nuclear medicine. Vaccine interventions involved the administration of vaccines for the prevention or treatment of disease.

We then grouped the trials according to target sample size, trial phase, sponsorship, and recruitment status. Target sample sizes were reported in the ICTRP as single numbers; these were grouped into six ranges based on the observed distribution in the dataset, with cut-points selected to reflect clinically meaningful thresholds: 4–20, 21–50, 51–100, 101–500, 501–1,000, and >1,000 participants. Trial phase was used directly from the ICTRP phase field. Trials recorded as ‘N/A’ in the phase field were retained as a separate ‘phase N/A’ category. Primary sponsorship was categorised into three groups: university/academic, industry, and other, using a keyword-based classification of sponsor names, followed by manual review of ambiguous entries. We note that the primary sponsor field reflects the registered administrative sponsor and may not fully capture the actual source of funding, as industry-funded studies may be registered under academic or hospital sponsors. Recruitment status was derived from the ICTRP status field and verified through manual check.

### Statistical analysis

We used Excel, version 16.112.1 (Microsoft, Redmond, Washington, USA) and Python, version 3.11.9 (Python Software Foundation, Wilmington, Delaware, USA) to descriptively summarise the key characteristics of the trials. Specifically, we managed the data and conducted basic statistical calculations in Excel, and used Python for more advanced data manipulation and visualisation, utilising the ‘pandas’ library for data processing and ‘matplotlib’ and ‘seaborn’ for figure generation.

## RESULTS

**Figure 1:**
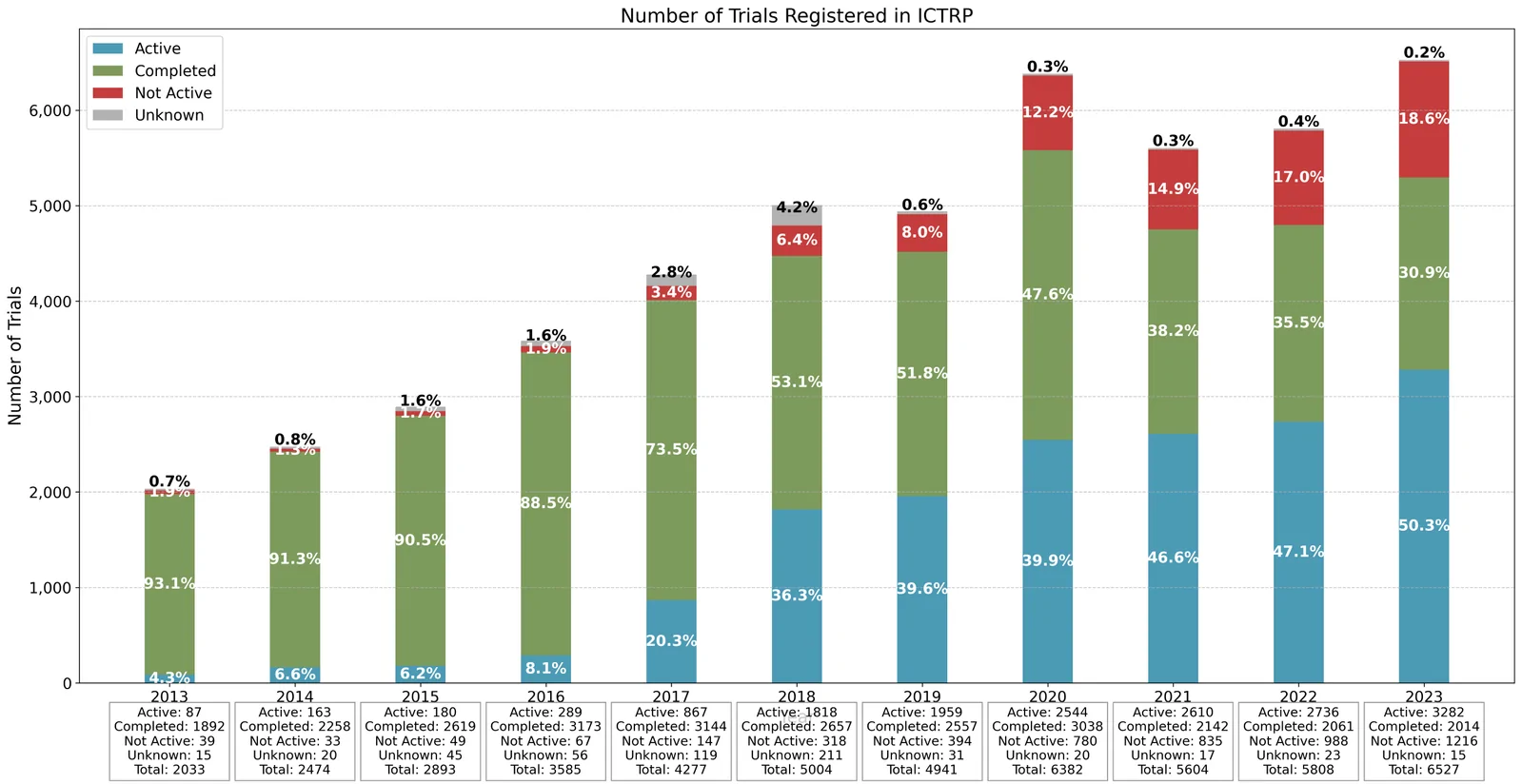
Number of trials registered by year and status in the ICTRP platform from 2013 to 2023 for EMR countries.

Of the 58,033 records retrieved from the ICTRP, no records were excluded because of missing registration dates. A total of 7,605 (13.1%) observational studies were excluded, leaving 50,428 (86.9%) interventional records. Following deduplication, 899 duplicate records were removed, resulting in a final analytic sample of 49,529 trials (**Figure 1**). We observed a substantial increase in the number of registered trials, from 2,033 in 2013 to 6,527 in 2023, representing an over three-fold increase over the decade. This upward trend was marked by a consistent rise in the number of trials from 2013 to 2018 and a notable peak in 2020, with 6,382 trials. The trend continued with a decrease in 2021, followed by a recovery in 2022 and 2023. The number of active trials increased dramatically from 87 (4.3%) in 2013 to 3,282 (50.3%) in 2023, while that of ‘not active’ trials grew from 39 (1.9%) in 2013 to 1,216 (18.6%) in 2023.

**Figure 2:**
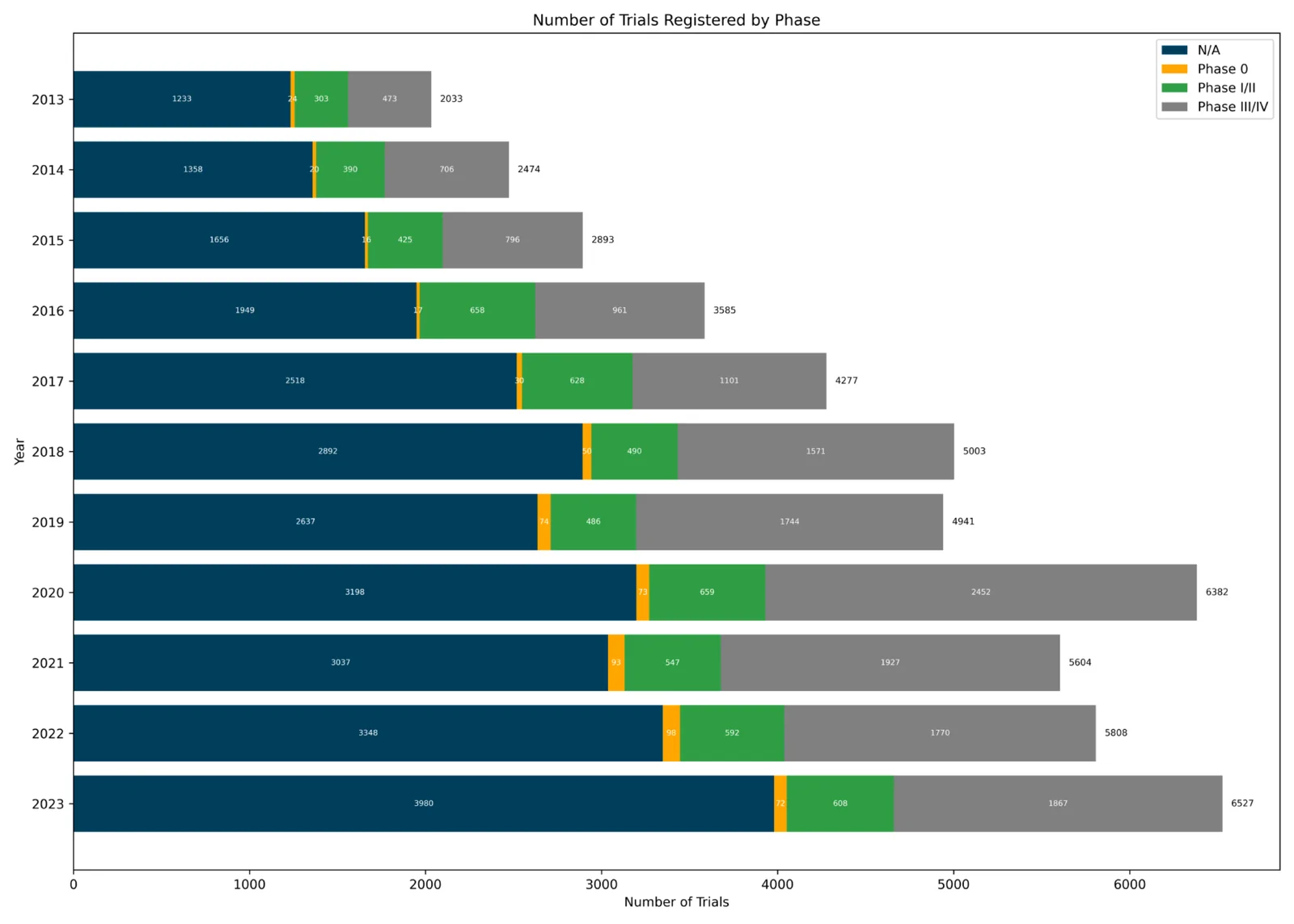
Number of trials registered by year and phase category.

Regarding trial phase distribution, phase N/A trials represented the largest category throughout the study period, accounting for 1,233 trials (60.6%) in 2013 and 3,980 (61.0%) in 2023, with proportions remaining stable across the decade. The number of phase III/IV trials showed notable absolute growth, increasing from 473 (23.3%) in 2013 to a peak of 2,452 (38.4%) in 2020, before dropping to 1,867 (28.6%) in 2023. The number of phase I/II trials fluctuated over the period, rising from 303 (14.9%) in 2013 to a peak of 659 (10.3%) in 2020, ending at 608 (9.3%) in 2023. Phase 0 trials remained consistently rare, never exceeding 2% in any year (**Figure 2**).

Overall, 33,292 trials (67.2%) enrolled adults only, 4,951 (10.0%) enrolled children only, and 11,284 (22.8%) included both adults and children. Regarding gender eligibility, 10,572 trials (21.3%) enrolled woman only and 2,640 (5.3%) enrolled men only. The proportion of university-sponsored trials remained relatively constant over the years, despite an increase from 1,780 (87.6%) in 2013 to 5,729 (87.9%) in 2023, while the industry-sponsored trials accounted for 2–3% of the whole sample. Small sample sizes predominated in the included trials, with over 70% having a target sample size of 100 or fewer participants (**Table 1**).

**Table 1:** Trial registration information by year, age, gender, sponsor type, and target sample size, n (%)

Year	Total	Adults	Children	Adults and Children	Men only	Women only	All	Industry	Other	University	4–20	21–50	51–100	101–500	501–1,000	>1,000
2013	2,033	1,221 (59.9)	208 (10.2)	604 (29.7)	121 (5.9)	496 (24.4)	1,416 (69.7)	38 (1.9)	215 (10.5)	1,780 (87.6)	132 (6.7)	458 (22.4)	964 (47.5)	430 (21.1)	16 (0.8)	13 (0.6)
2014	2,474	1,507 (60.9)	238 (9.6)	729 (29.4)	115 (4.6)	624 (25.2)	1,735 (70)	47 (1.9)	261 (10.5)	2,166 (87.4)	125 (5.0)	519 (20.6)	1,164 (46.9)	585 (23.6)	29 (1.2)	22 (0.9)
2015	2,893	1,807 (62.4)	277 (9.6)	809 (27.9)	138 (4.8)	773 (26.7)	1,982 (68.4)	55 (1.9)	326 (11.2)	2,512 (86.9)	155 (5.4)	534 (18.5)	1,450 (50.1)	682 (23.6)	21 (0.7)	15 (0.5)
2016	3,585	2,256 (63)	327 (9.1)	1,002 (28)	183 (5.1)	958 (26.7)	2,444 (68.2)	77 (2.1)	367 (10.2)	3,141 (87.5)	215 (4.6)	760 (21.6)	1,742 (48.5)	770 (21.5)	31 (0.9)	19 (0.5)
2017	4,277	2,734 (63.8)	425 (9.9)	1,118 (26.1)	229 (5.4)	1,120 (26.2)	2,928 (68.4)	63 (1.5)	450 (10.5)	3,764 (87.8)	238 (4.6)	933 (21.8)	2,089 (48.9)	899 (21.0)	32 (0.7)	29 (0.7)
2018	5,004	3,275 (65.4)	546 (10.9)	1,182 (23.6)	257 (5.1)	1,290 (25.8)	3,456 (69.1)	48 (1)	510 (10.2)	4,446 (88.7)	321 (4.4)	1,134 (22.7)	2,438 (48.7)	990 (19.8)	54 (1.1)	32 (0.6)
2019	4,941	3,317 (67.1)	467 (9.5)	1,157 (23.4)	267 (5.4)	1,070 (21.6)	3,604 (72.8)	74 (1.5)	428 (8.6)	4,439 (89.9)	334 (4.0)	1,272 (25.7)	2,344 (47.4)	926 (18.7)	21 (0.4)	14 (0.3)
2020	6,382	4,419 (69.1)	635 (9.9)	1,328 (20.8)	336 (5.3)	1,182 (18.5)	4,864 (76.2)	137 (2.1)	633 (9.9)	5,612 (87.9)	485 (4.5)	1,709 (26.7)	2,932 (46.0)	1,127 (17.6)	43 (0.7)	31 (0.5)
2021	5,604	3,977 (71)	550 (9.8)	1,077 (19.2)	296 (5.3)	953 (17)	4,355 (77.7)	214 (3.8)	510 (9.1)	4,880 (87.1)	446 (4.8)	1,619 (28.8)	2,528 (45.7)	931 (16.6)	35 (0.6)	35 (0.6)
2022	5,809	4,128 (71.1)	576 (9.9)	1,104 (19)	310 (5.3)	943 (16.2)	4,555 (78.4)	212 (3.7)	538 (9.3)	5,058 (87.0)	479 (4.0)	1,806 (30.8)	2,575 (44.2)	895 (15.4)	32 (0.6)	18 (0.3)
2023	6,527	4,651 (71.3)	702 (10.8)	1,174 (17.9)	388 (5.9)	1,163 (17.8)	4,976 (73.1)	183 (2.8)	615 (9.4)	5,729 (87.9)	537 (4.8)	2,126 (32.6)	2,815 (43.2)	997 (15.3)	33 (0.5)	19 (0.3)
Grand total	49,529	33,292 (67.2)	4,951 (10.0)	11,284 (22.8)	2,640 (5.3)	10,572 (21.3)	36,315 (73.1)	1,148 (2.3)	4,853 (9.8)	43,527 (87.7)	3,467 (7.0)	12,870 (24.9)	23,041 (46.5)	9,232 (17.7)	347 (0.7)	247 (0.5)

Pharmacological interventions comprised 27,402 (55.3%) of all trials. Their absolute number increased from 1,251 (61.5% of trials registered in 2013) to 2,768 (42.4% of trials registered in 2023), while their proportional share decreased over the study period (**Table 2**). Physical interventions showed substantial growth, increasing from 11% to 30.6%. Behavioural interventions remained relatively stable, accounting for 14.1% of total trials. Device interventions doubled their share from 3.4% to 7.0% over the period. Biological, herbal, radiation, and vaccine interventions accounted for 1.6%, 3.3%, 0.5%, and 0.3% of all trials, respectively. Randomised trials constituted a consistently high proportion of registered trials throughout the study period, accounting for 87% in 2013 and 87.3% in 2023, and 86.3% of all trials overall.

**Table 2:** Trial intervention type and allocation information by year, n (%)

Year	Total	Behavioural	Biological	Device	Herbal	Pharmacological	Physical	Radiation	Vaccine	Non-randomised	Randomised	Unknown
2013	2,033	402 (19.7)	21 (1.03)	70 (3.44)	56 (2.75)	1,251 (61.5)	224 (11)	3 (0.15)	6 (0.29)	65 (3.2)	1,778 (87)	198 (9.7)
2014	2,474	429 (17.3)	21 (0.84)	89 (3.60)	56 (2.26)	1,602 (64.7)	259 (10.5)	10 (0.40)	8 (0.32)	113 (4.56)	2,116 (85.5)	245 (9.9)
2015	2,893	435 (15.03)	28 (0.96)	94 (3.25)	73 (2.52)	1,893 (65.4)	347 (11.99)	13 (0.45)	10 (0.35)	121 (4.2)	2,504 (86.5)	268 (9.3)
2016	3,585	556 (15.5)	45 (1.25)	119 (3.32)	113 (3.15)	2,269 (63.3)	452 (12.61)	17 (0.47)	14 (0.39)	175 (4.9)	3,110 (86.7)	299 (8.3)
2017	4,277	664 (15.5)	39 (0.91)	156 (3.64)	141 (3.29)	2,434 (56.90)	813 (19.0)	22 (0.51)	8 (0.18)	239 (5.6)	3,714 (86.8)	324 (7.6)
2018	5,004	927 (18.5)	90 (1.8)	186 (3.72)	160 (3.20)	2,543 (50.82)	1,077 (21.5)	34 (0.68)	11 (0.22)	370 (7.4)	4,253 (84.9)	380 (7.5)
2019	4,941	685 (13.9)	90 (0.18)	219 (4.43)	130 (2.63)	2,819 (57)	956 (19.3)	28 (0.56)	14 (0.28)	350 (7.1)	4,221 (85.5)	370 (7.4)
2020	6,382	666 (10.44)	137 (2.15)	337 (5.28)	200 (3.13)	3,702 (58)	1,288 (20.2)	32 (0.5)	20 (0.31)	449 (7)	5,426 (85.1)	507 (7.9)
2021	5,604	653 (11.65)	96 (1.71)	315 (5.62)	204 (3.64)	3,071 (54.8)	1,199 (21.4)	27 (0.48)	39 (0.7)	322 (5.7)	4,887 (87.1)	395 (7)
2022	5,809	665 (11.45)	118 (2.03)	360 (6.2)	228 (3.92)	3,050 (52.5)	1,334 (23)	31 (0.53)	22 (0.38)	344 (5.9)	5,045 (86.8)	419 (7.2)
2023	6,527	891 (13.65)	115 (1.76)	459 (7.03)	257 (3.93)	2,768 (42.4)	1,995 (30.56)	36 (0.55)	6 (0.09)	366 (5.6)	5,697 (87.3)	464 (7.1)
Grand total	49,529	6,973 (14.1)	775 (1.56)	2,404 (5)	1,618 (3.3)	27,402 (55.3)	9,944 (20.1)	253 (0.5)	158 (0.3)	2914 (5.9)	42,743 (86.3)	3,869 (7.8)

**Figure 3:**
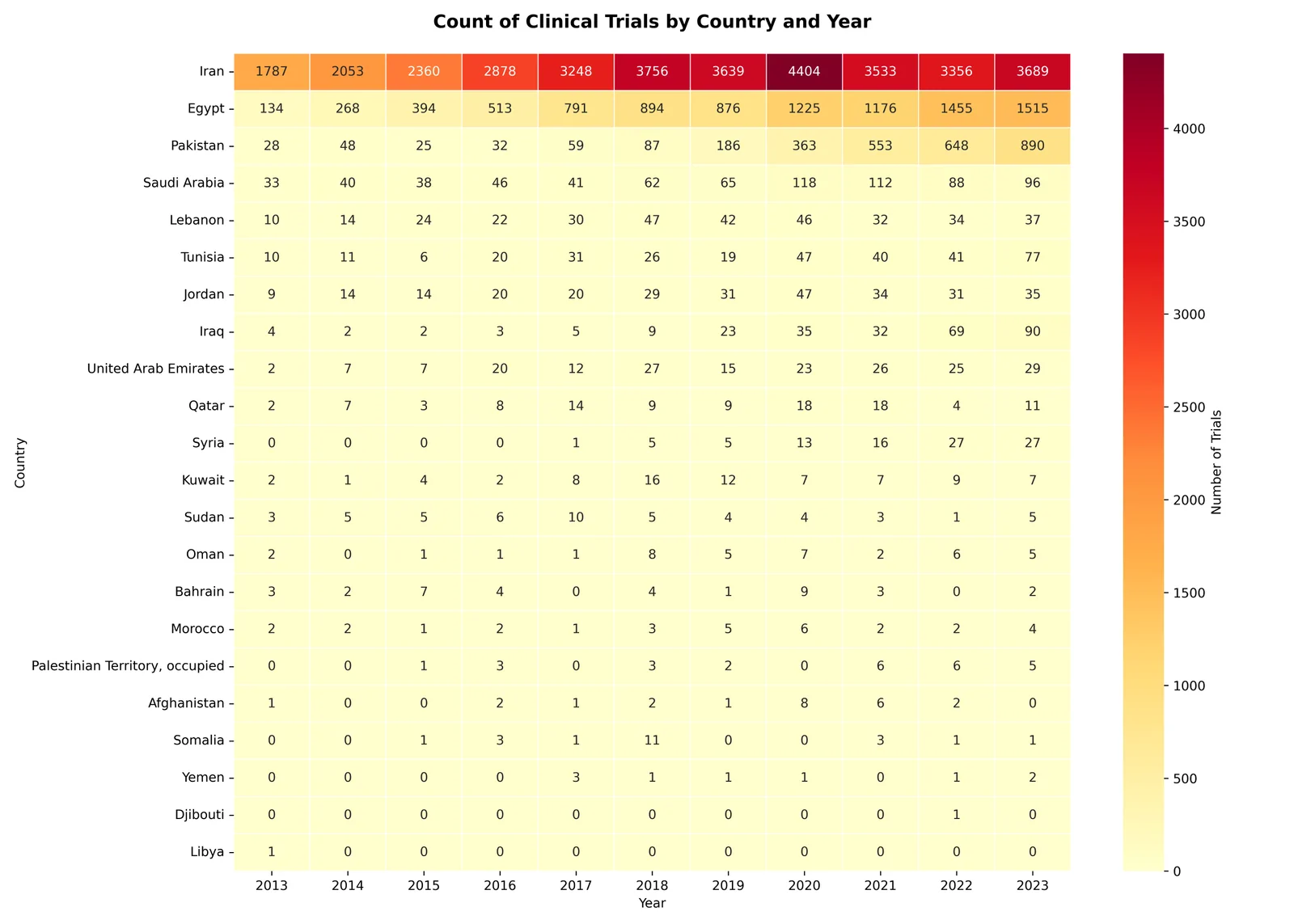
Annual count of clinical trials registered by country from 2013 to 2023.

Iran (n = 34,703, 70.1%) and Egypt (n = 9,240 trials, 18.7%) were the most active contributors to the trials in our sample. While both countries saw increases in the number of trials in the study period (**Figure 3**), Egypt showed a notable 10-fold increase (from 134 to 1,515) as compared to Iran’s two-fold increase (from 1,787 to 3,689). The two countries were followed by Pakistan, Saudi Arabia, Iraq, and Tunisia (890, 96, 90, and 77 trials in 2023, respectively). Very few trials were registered from Djibouti (n = 1), Libya (n = 1), Yemen (n = 10), Somalia (n = 21), Afghanistan (n = 23), and occupied Palestinian territories (n = 25).

## DISCUSSION

This first analysis of trials registered in the ICTRP for the countries of the EMR highlights both progress and persistent challenges in the region’s clinical research landscape. The 221% increase in the number of registered trials from 2013 to 2023 represents a substantial growth of clinical research activity, reflecting increasing research capacity and a growing recognition of the importance of clinical trials. This trend aligns with global efforts to enhance clinical research capacity in developing regions [11]. The notable peak in 2020 (6,382 trials) likely reflects a surge in COVID-19-related research, demonstrating the region’s ability to respond rapidly to global health emergencies, akin to the patterns observed globally during the pandemic [12].

We also observed potential challenge in research governance, as a substantive number of trials remained incomplete over the period. While this might be understandable for more recently initiated trials, it might be less explainable for 20% of the trials registered in 2017 and 36% of those registered in 2018. It is also concerning that 31.8% were registered retrospectively.

Another key observation was the predominance of trials with small sample sizes: less than 20% of all trials had a target sample size of over 100 participants and only 1.2% targeted over 500 participants. Notably, such trials seem to have become more common over time. Certain trials, including pilot studies, feasibility investigations, and early-phase studies, may appropriately require small sample sizes. However, without stratification by phase, intervention type, and study design, we cannot determine what proportion of small trials reflect appropriate designs, rather than a lack of statistical power. Nevertheless, the near absence of large-scale trials remains a concern and may be a key consideration for the promotion of regulatory approaches and collaborative practices that will encourage such studies in the future.

### Regional disparities and research concentration

We noted a significant geographical concentration of clinical trials within a few countries of the region, with Iran and Egypt accounting for close to 90% of all trials. Although the pattern, in general, is in line with the geographical distribution of overall biomedical and health research output in the EMR [3,13], the concentration is more pronounced for clinical trials. Whether this is related to regulatory capacity, technical expertise, or the academic value given to interventional studies warrants further research [14].

The consistently high proportion of phase N/A trials throughout the study period – approximately 60% in both 2013 and 2023 – reflects the broad scope of the ICTRP interventional category. Trails recorded as ‘phase N/A’ refer to studies that do not follow national regulatory agencies-defined phases for product approval and registration, and may cover nursing care, behavioural interventions, service delivery, or comparative assessment of existing treatment approaches [15]. This high proportion may also reflect incomplete phase reporting across registries or inconsistent application of phase definitions across countries and institutions.

Most trials (73.1%) were open to all genders, while women-only trials accounted for 21.3% and men-only trials for 5.3% of the sample. Given the global concern regarding the underrepresentation of women in clinical trials [16], these data alone do not allow us to assess whether they were adequately represented among participants enrolled in the trials in the EMR. Similarly, although trials enrolling adults only constituted the largest age category, this finding alone does not allow us to assess whether paediatric populations are adequately represented relative to regional disease burden and research needs.

The distribution of clinical trial sponsorship points to the EMR’s complex research ecosystem. While universities sponsored 87.7% of all trials, demonstrating a robust academic research capacity in countries with established educational institutions, industry sponsorship accounted for a low 2.3% of the sample. However, this apparent disparity may not fully reflect actual commercial engagement, as many industry trials might operate under university affiliations.

Our findings have two important implications for health policy and research development in the EMR. First, targeted capacity-building initiatives are needed in countries with limited trial activity. Second, maintaining research momentum beyond crisis-driven peaks and unlocking new opportunities for increasing industry involvement and research funding diversification also remains important.

Important limitations should be considered when interpreting these findings. Our analysis is based on self-reporting of the research plans to accredited registries, which might not have been updated as needed when trials have moved between statuses. The ICTRP collates the data from the registries, so limitations and variations within individual registries might have affected the completeness and validity of the data. To this point, the dataset contained inconsistencies in registration formats, missing values, and non-standardised entry methods across different countries and institutions, which may have influenced our ability to accurately capture and analyse trends. Lastly, as most of the trials originated from a few countries, our findings should be interpreted in terms of the national capacities and regulatory framework of those countries, with care in generalisation to other contexts.

## CONCLUSIONS

The analysis of 49,529 clinical trials in the EMR from 2013 to 2023 points to a complex and evolving research landscape. While the 221% increase in the number of registered trials shows a substantial growth in research capacity, significant challenges persist: most trials were conducted in just six countries, with universities remaining the primary sponsors and with industry having a limited role.

These findings emphasise a need for targeted interventions to strengthen the research infrastructure in underrepresented countries, enhance frameworks for regional collaboration and multi-centre multi-country trials, and increase industry participation in research. Success in developing the EMR’s clinical trial landscape will require coordinated efforts to address these challenges, while ensuring a balanced distribution of research opportunities across all countries in the region. Our study can only be understood as a foundational mapping exercise; deeper analyses of therapeutic priorities, multi-country collaboration, publication outcomes, and alignment with regional disease burden are needed in the future.

## Data Availability

**Data availability:** All data used in this study are publicly available from the WHO ICTRP: https://www.who.int/clinical-trials-registry-platform

## References

[1] World Health Organization. Strengthening clinical trials. Available: https://www.who.int/our-work/science-division/research-for-health/implementation-of-the-resolution-on-clinical-trials. Accessed: 10 August 2026.

[2] Sirleaf EJ Clark H Report of the Independent Panel for Pandemic Preparedness and Response: making COVID-19 the last pandemic. Lancet. 2021;398:101–3. 10.1016/S0140-6736(21)01095-3 33991477 PMC9751704

[3] Tadmouri GO Mandil A Soukarieh I Salma I Assaad N Al Khatib M Analysis of biomedical and health research in the Eastern Mediterranean Region. East Mediterr Health J. 2024;30:414–23. 10.26719/2024.30.6.414 39545291

[4] Rashidian A Wu K Al Ariqi L Aly E Mandil A Barakat A WHO’s support for COVID-19 research and knowledge management in the Eastern Mediterranean Region. BMJ Glob Health. 2022;7:e008737. 10.1136/bmjgh-2022-008737 35750342 PMC9226463

[5] Karimian Z Bellizzi S Sirous S Lukwiya M Ahmed A Fahmy K Shaping local health policies during health emergencies: insights from WHO COVID-19 vaccine effectiveness study in the Eastern Mediterranean Region. East Mediterr Health J. 2024;30:539–41. 10.26719/2024.30.8.539 39559970

[6] World Health Organization. International Clinical Trials Registry Platform (ICTRP). Available: https://www.who.int/tools/clinical-trials-registry-platform. Accessed: 10 August 2026.

[7] Feizabadi M Fahimnia F Mosavi Jarrahi A Naghshineh N Tofighi S Iranian clinical trials: An analysis of registered trials in International Clinical Trial Registry Platform (ICTRP). J Evid Based Med. 2017;10:91–6. 10.1111/jebm.12248 28444844

[8] Siddiqui A Zafar A Irfan T Comprehensive analysis of clinical trials in Pakistan from September 1992 to February 2019. J Ayub Med Coll Abbottabad. 2022;34:468–73. 10.55519/JAMC-03-8793 36377158

[9] Al-Hajri A Al-Khabori M Rasool W Productivity of clinical trials conducted in the Gulf Cooperative Council Region. Sultan Qaboos Univ Med J. 2022;22:501. 10.18295/squmj.11.2021.144 36407711 PMC9645501

[10] Odedina FT Shamley D Okoye I Ezeani A Ndlovu N Dei-Adomakoh Y Landscape of oncology clinical trials in Africa. JCO Glob Oncol. 2020;6:932–41. 10.1200/JGO.19.00189 32614728 PMC7392757

[11] Council for International Organizations of Medical Sciences Working Group on Clinical Research in Resource-Limited Settings. Clinical research in resource-limited settings. Geneva, Switzerland: Council for International Organizations of Medical Sciences (CIOMS); 2021.

[12] Janiaud P Axfors C Van’t Hooft J Saccilotto R Agarwal A Appenzeller-Herzog C The worldwide clinical trial research response to the COVID-19 pandemic - the first 100 days. F1000 Res. 2020;9:1193. 10.12688/f1000research.26707.2 33082937 PMC7539080

[13] World Health Organization Regional Office for the Eastern Mediterranean. Health research in the Eastern Mediterranean Region: current status and challenges. Cairo, Egypt: World Health Organization Regional Office for the Eastern Mediterranean; 2020. Available: https://iris.who.int/items/8f48e740-c50e-4d43-be7a-5800741dd141. Accessed: 10 August 2026.

[14] Ismail SA McDonald A Dubois E Aljohani FG Coutts AP Majeed A Assessing the state of health research in the Eastern Mediterranean Region. J R Soc Med. 2013;106:224–33. 10.1258/jrsm.2012.120240 23761582 PMC3705424

[15] Toolkit. Phases of clinical trials. Available: https://toolkit.ncats.nih.gov/glossary/phases-of-clinical-trials. Accessed: 10 August 2026.

[16] Daitch V Turjeman A Poran I Tau N Ayalon-Dangur I Nashashibi J Underrepresentation of women in randomised controlled trials: a systematic review and meta-analysis. Trials. 2022;23:1038. 10.1186/s13063-022-07004-2 36539814 PMC9768985

